# The effect of intravitreal bevacizumab in a rare case of retinal dystrophy with secondary cystoid macular edema

**DOI:** 10.22336/rjo.2017.23

**Published:** 2017

**Authors:** Mioara-Laura Macovei, Maria-Alexandra Nica

**Affiliations:** * Ophthalmology Department, “Dr. Carol Davila” Central Military Emergency University Hospital, Bucharest, Romania

**Keywords:** Retinitis punctata albescens, anti-VEGF, cystoid macular edema

## Abstract

The authors presented a clinical case of retinitis punctate albescens in a 26-year-old female patient, with a family history of typical retinitis pigmentosa (father) and bilateral cystoid macular edema treated with anti-VEGF (bevacizumab).

## Introduction

This disorder is often considered to belong to the category of retinal diseases known as flecked retina syndrome. Retinitis punctata albescens (RPA) is a rare form of non-syndromic, generally referred to as a subtype of autosomal recessive retinitis pigmentosa with specific characteristics.

RPA is an inherited retinal dystrophy characterized by progressive night blindness and presence of small white dots on the retina. Furthermore, the scotopic ERG waveforms usually do not regenerate. RPA prevalence is currently estimated at 1/800 000 people [**1**,**3**].

## Material and methods – Case report

A 26-year-old female accidentally presented in our clinic, along with her father, previously known with typical retinitis pigmentosa, who was scheduled for cataract surgery. Having established the diagnosis, an ophthalmological exam was suggested for the daughter. The patient history revealed that she had been suffering from night blindness ever since and decreased vision for a few months. However, she had never addressed an ophthalmologist. 

At presentation, her best-corrected visual acuity was RE: 0.6 nc, LE: 0.4 nc. The IOP was normal in both eyes BE IOP: 13 mmHg on non-contact tonometry.

**Fig. 1:**
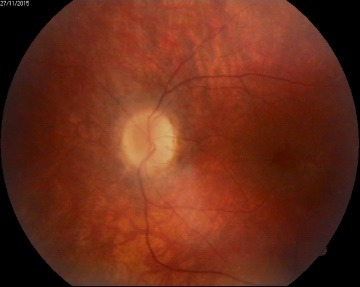
*Attenuation and narrowing of the retinal vessels,*retinal atrophy, *waxy pallor of the optic disc, *absence of foveal reflex with intraretinal cystic areas

**Fig. 2:**
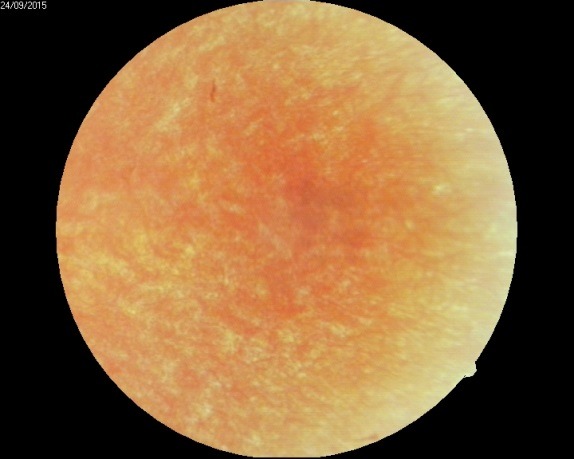
*Typical small white dot-like spots in the periphery

Slit-lamp examination of the anterior segment revealed no abnormal findings. In order to examine the posterior segment (**Fig. 1**,**2**), pharmaceutical mydriasis with 0.5% tropicamide and 1% cyclopentolate ophthalmic solutions was used. 

**Fig. 3:**
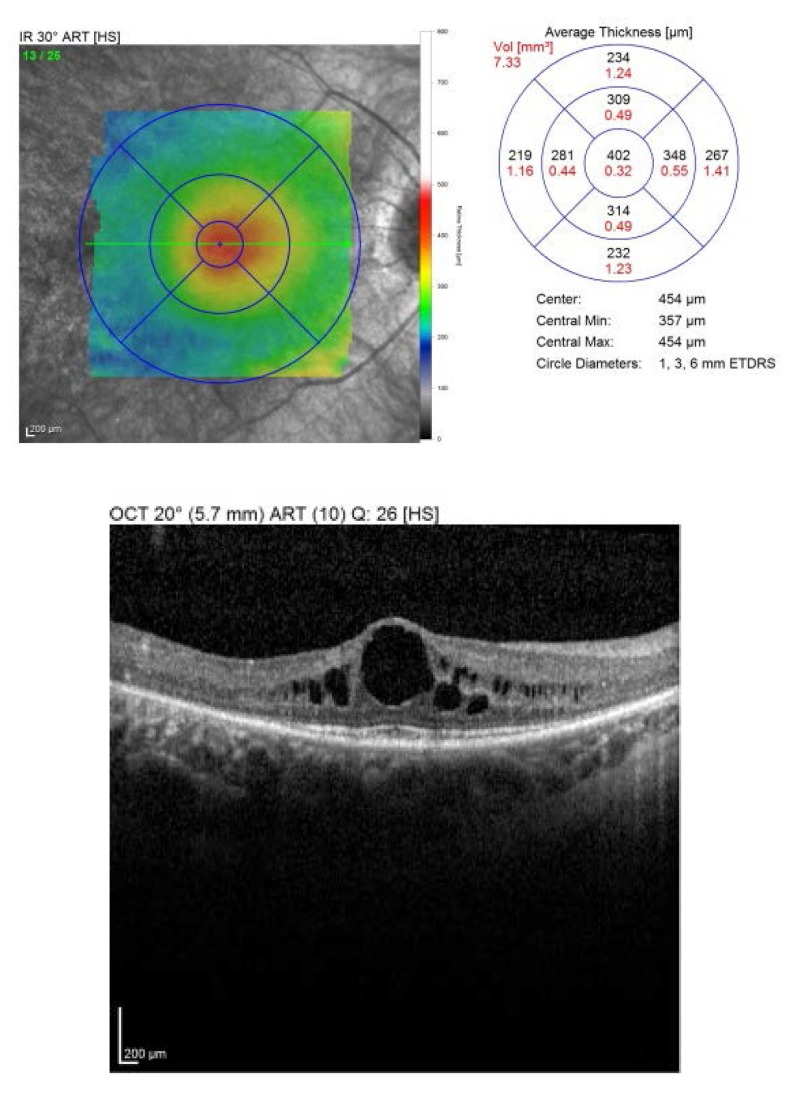
*Macular OCT of the right eye

**Fig. 4:**
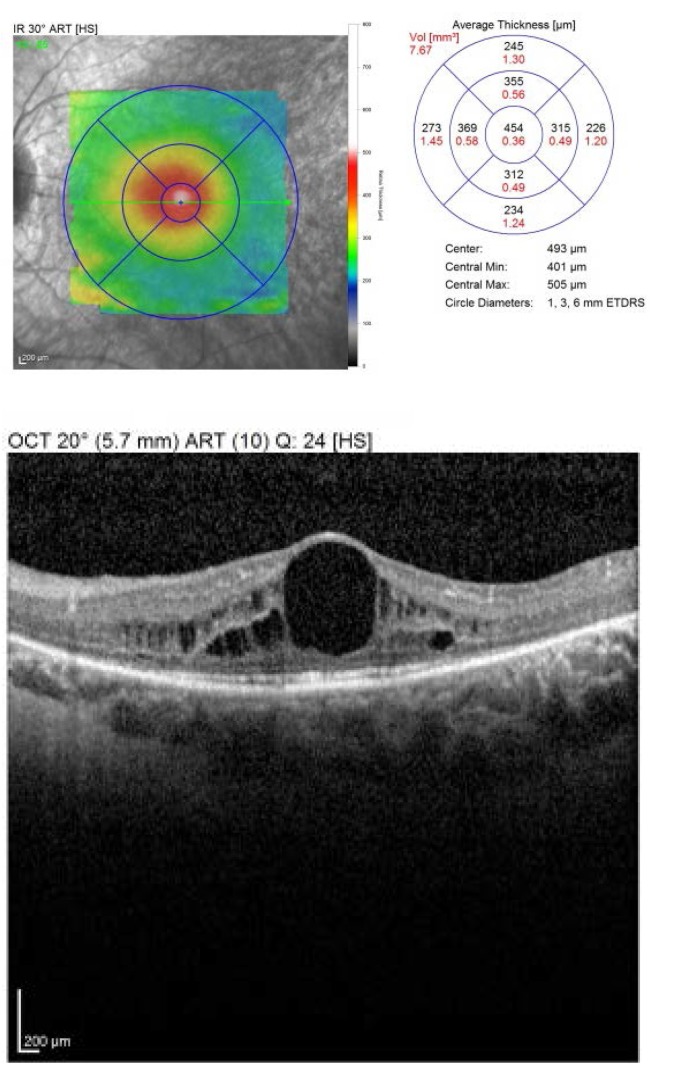
*Macular OCT of the left eye

The OCT showed an increased retinal thickens with hyporeflective cysts, suggestive for CME. Fluorescein angiography was proposed, but refused by the patient (**Fig. 3**,**4**).

**Fig. 5:**
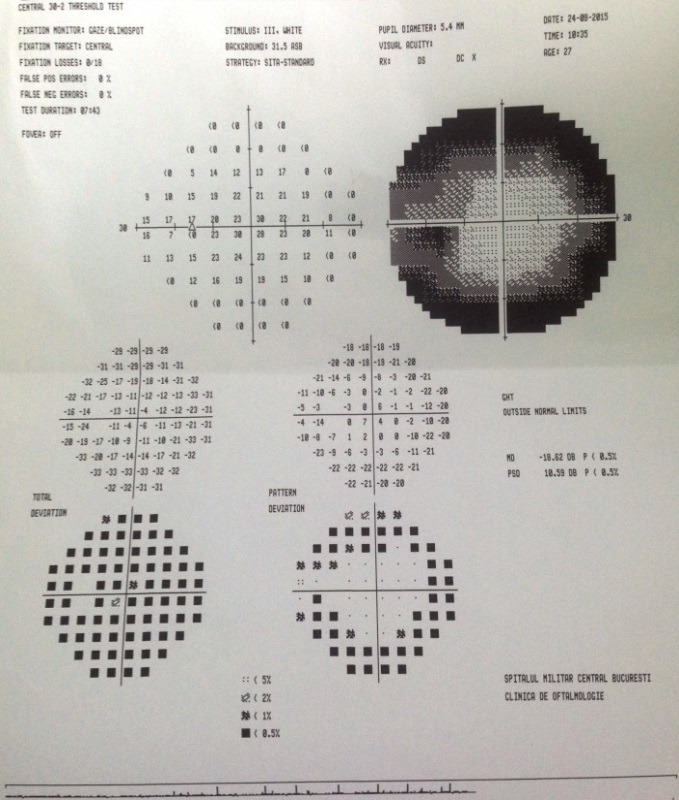
*Visual field of the right eye

**Fig. 6:**
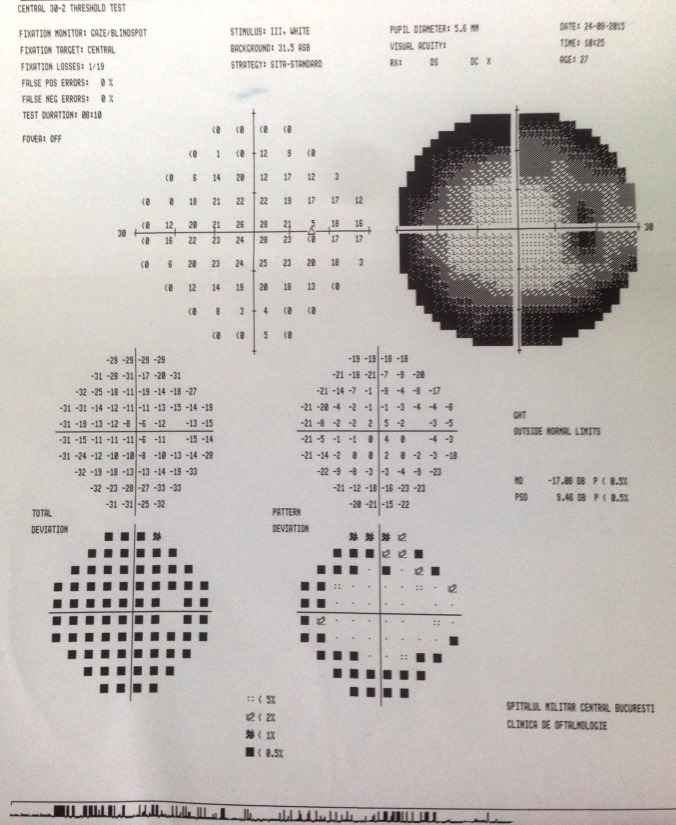
*Visual field of the left eye

The visual fields revealed ring scotoma in both eyes, with a preserved central vision (mild tunnel vision) (**Fig. 5**,**6**).

**Fig. 7:**
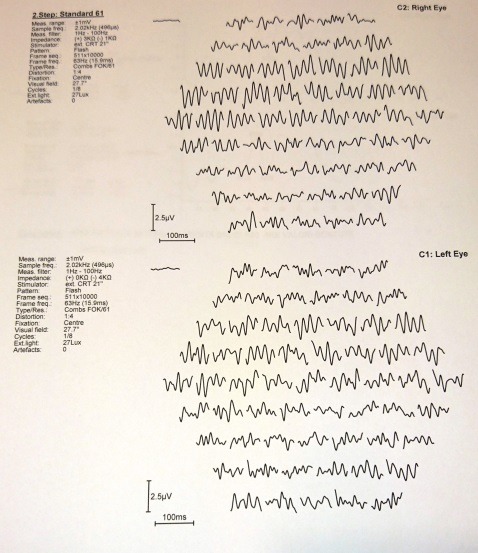
*Scotopic and photopic electroretinogram

**Fig. 8:**
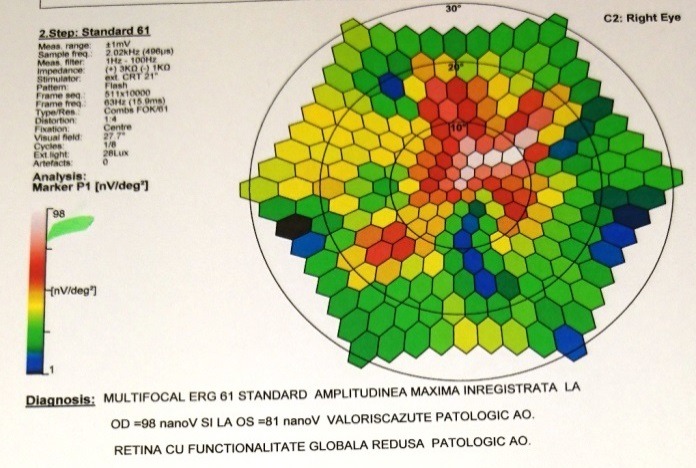
*Multifocal electroretinogram of the right eye

**Fig. 9:**
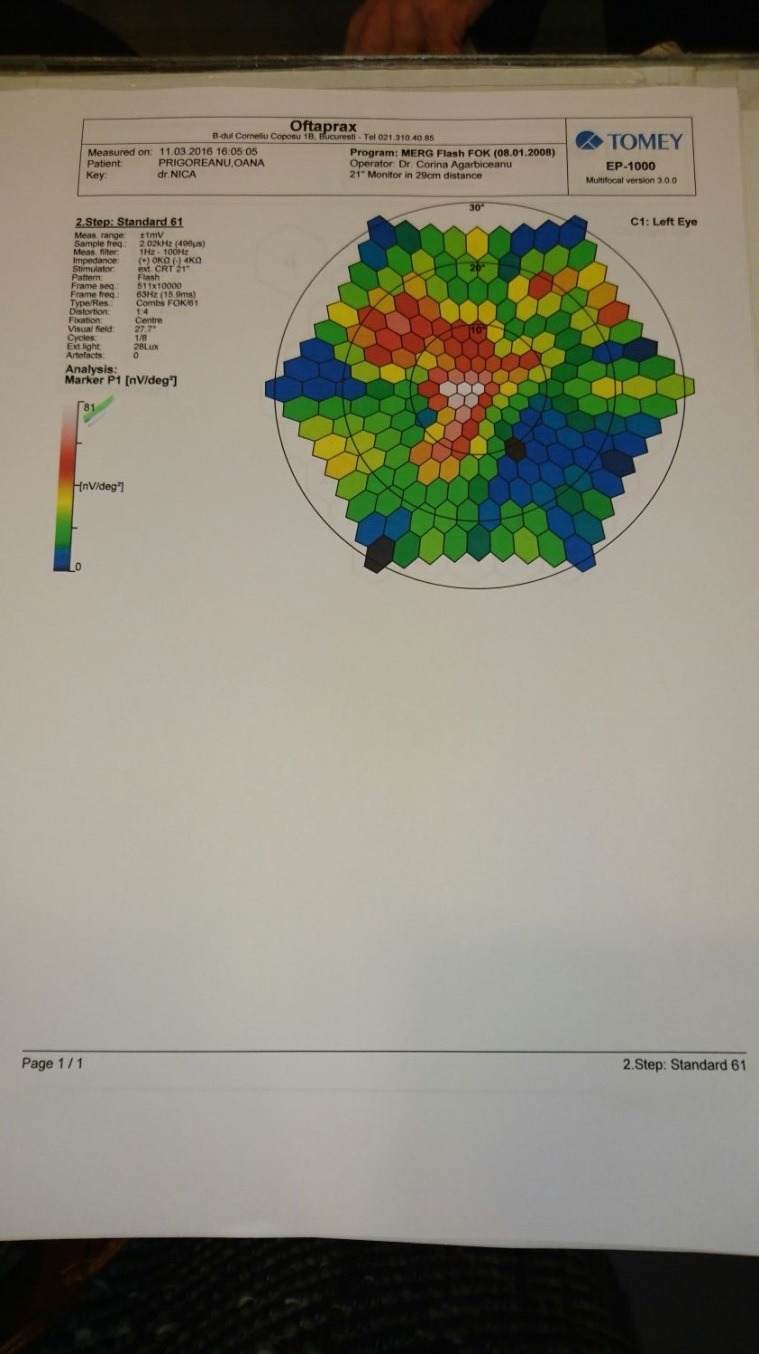
*Multifocal electroretinogram of the left eye

The electroretinogram (ERG) presents dramatic diminution in a- and b- waves’ amplitudes (**Fig. 7**), first the scotopic system (rods) is affected and then the photopic system (cones). The multifocal electroretinogram (mfERG) (**Fig. 8**,**9**) showed significant reductions in response to the amplitudes in the macular areas and a higher but also inappropriately lower response amplitude at the fovea.

The diagnosis of Retinitis Punctate Albescens with Cystoid Macular Edema in both eyes was established based on the family history, clinical findings, and investigations.

Treatment with topical dorzolamide, BE, was started for three times per day, oral acetazolamide 500 mg daily, and aspacardin 100 mg daily. After 3 days, the patient presented with dizziness, paresthesia, and palpitations [**2**]. The oral treatment was stopped and other therapeutic options were discussed: intravitreal Triamcinolone acetonide, although studies revealed insignificant effects [**4**,**6**], and intravitreal anti-VEGF agents, with recent studies demonstrating an effect in lowering fluid accumulation [**5**]. 

Bevacizumab 1,25mg/ 0,05 ml was injected in both eyes, one week apart.

**Fig. 10:**
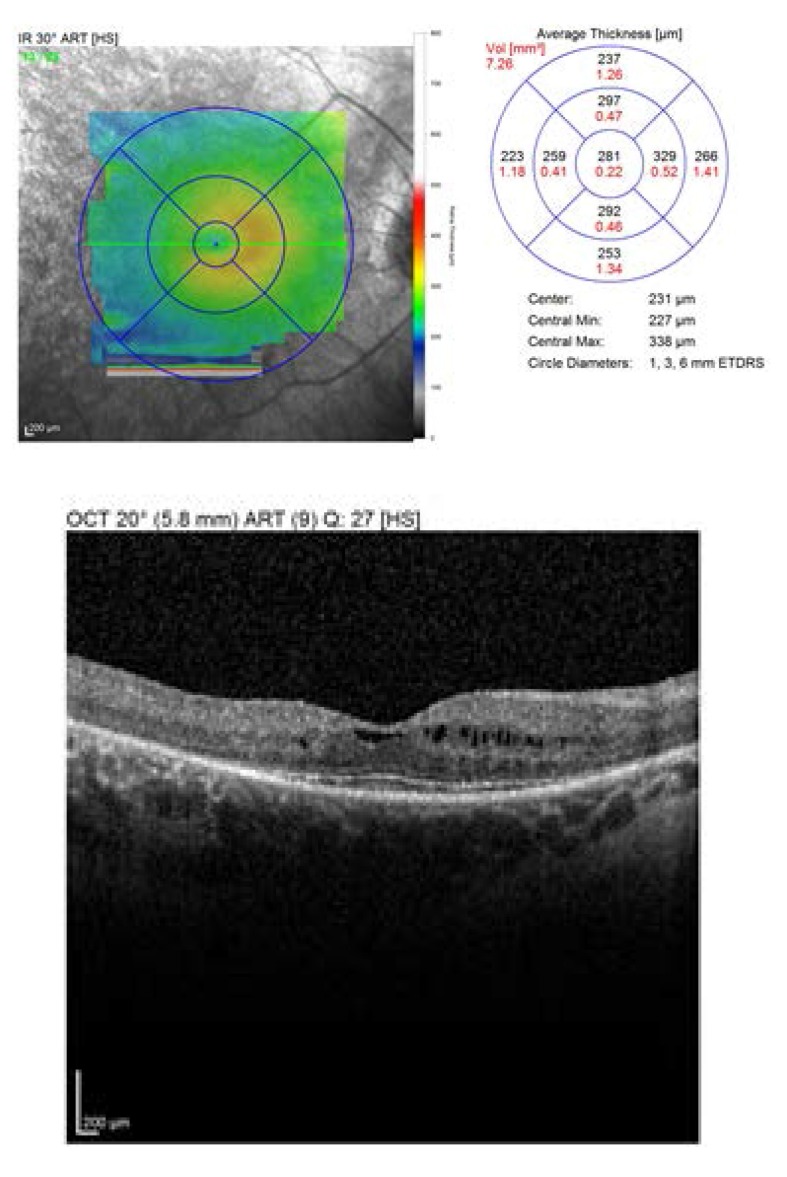
*Macular OCT of the right eye

**Fig. 11:**
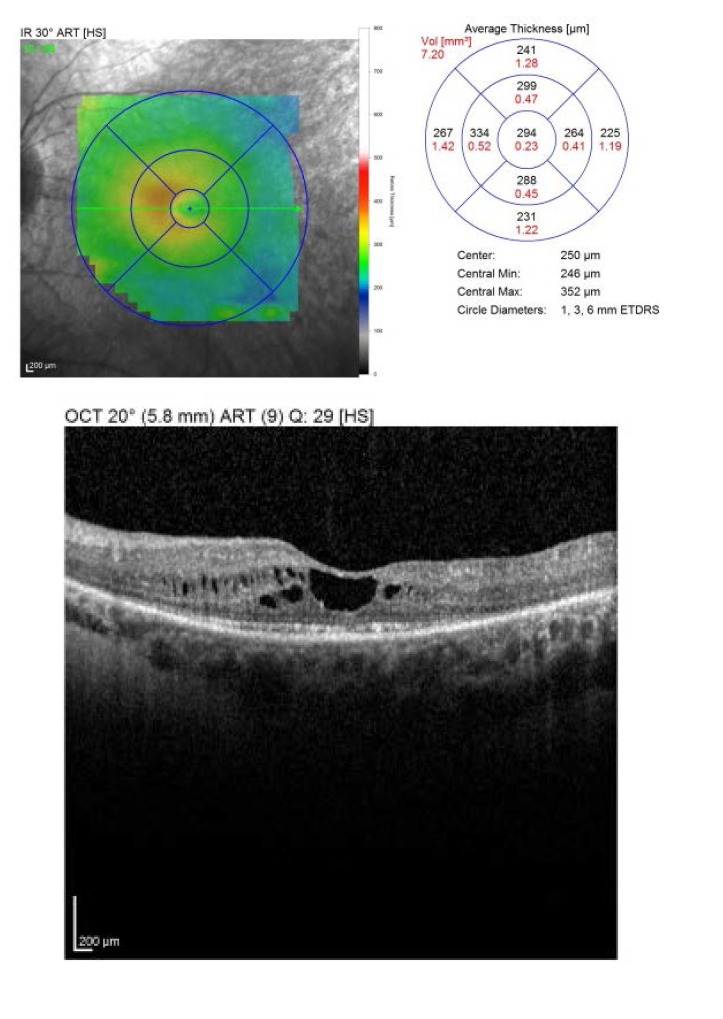
*Macular OCT of the left eye

2 weeks later, at the first follow-up, the BCVA was RE: 1, LE: 0.8 nc. The OCT revealed a central retinal thickness of RE: 281 µm (**Fig. 10**), LE: 294 µm (**Fig. 11**), a significant decrease, from about 400 and 450 µm previously. She was followed-up monthly for the next 6 months, presenting a stable evolution. She is expected for further examinations at every 6 months or if a loss in visual acuity is experienced.

## Discussion

• RPA is a rod-cone dystrophy, a subtype of RP; so far, there has been no available treatment; however, the correct diagnosis provides the basis for counseling school or career choices;

• Genetic testing provides important information for prognosis, as well as risk assessment to family members. The National Eye Institute (NEI) is currently creating the eyeGENE Network, which aids in the recruitment of patients interested in participating in future clinical trials related to genetic eye diseases.

• On long term, we expect the narrowing of visual fields, photophobia, decrease in central vision leading to irreversible blindness.

• The particularity of this case is that the patient presented accidentally in our clinic, without having had a previous ophthalmologic exam, although having had night blindness and a family history of typical RP; macular involvement is frequent, usually as progressive macular atrophy, rarely with CME;

• Usually, oral and topical carbonic anhydrase inhibitors deliver good results in RPA with secondary CME; however, due to unpleasant side effects, intravitreal Bevacizumab was administered, with favorable results on short and medium term.

**Financial Disclosures**

The authors have no financial interests to disclose.
