# ERRATA

**Published:** 2017

The article "**The effect of intravitreal bevacizumab in a rare case of retinal dystrophy with secondary cystoid macular edema**", authors Macovei Mioara-Laura, Nica Maria-Alexandra, was published in Romanian Journal of Ophthalmology, 2017; 61(2): 123-127. The authors would like to change the text “oral acetazolamide 500 mg daily and aspacardin 100 mg daily” into **“Acetazolamida Arena oral 250mg x 2/ daily, Aspacardin Terapia oral 39mg/ 12mg x2/ daily”**.

The above-mentioned change should be made at all the levels, starting with the journal’s web page – www.rjo.ro and ending with the database the journal is indexed in (PubMed). 

**The publishers regret any inconvenience caused by this change**.

